# Pre-operative echocardiogram in hip fracture patients with cardiac murmur- an audit

**DOI:** 10.1186/1749-799X-6-49

**Published:** 2011-09-23

**Authors:** Prithee Jettoo, Rajesh Kakwani, Shahid Junejo, Imtiyaz Talkhani, Paul Dixon

**Affiliations:** 1 Department of Trauma and Orthopaedics, Sunderland Royal Hospital, Sunderland SR4 7TP, UK

## Abstract

**Background:**

All hip fracture patients with a cardiac murmur have an echocardiogram as a part of their preoperative work-up in our unit. We performed a retrospective audit to assess the impact of obtaining a pre-operative echocardiogram on the management of hip fracture patients.

**Methods:**

All hip fracture patients (N = 349) between 01/06/08 and 01/06/09 were included in the study. 29 patients had pre-operative echocardiogram (echo group). A computer generated randomised sample of 40 patients was generated from N, 'non-echo' group. Data was obtained from medical records and the Hospital Information Support System (HISS). The groups were compared using Student's t test. Approval was obtained locally from the clinical governance department for this project.

**Results:**

Age and gender distribution were similar in both groups. Indication for echo was an acute cardiac abnormality in 4 cases. 25 patients had echo for no new cardiac problem (indication being cardiac murmur in 23 patients and extensive cardiac history in 2 cases). Cardiology opinion was sought in 5 cases. No patient required cardiac surgery or balloon angioplasty preoperatively. Patients having pre-operative echo had significant delay to surgery (average 2.7 days, range 0-6 days) compared to 'non-echo' group (average 1.1 days, range 0-3 days), (p < 0.001). There was no significant difference in length of stay (p = 0.14) and mortality at 30 days (p = 0.41) between the groups.

**Conclusion:**

We have developed departmental guidelines for expediting echo requests in hip fracture patients with cardiac murmur. A liaison has been established with our cardiology department to prioritise such patients on the Echocardiography waiting list, to prevent unnecessary avoidable delay. Careful patient selection for pre-operative echocardiography is important to avoid unnecessary delay to surgery.

## Introduction

The incidence of hip fractures in the elderly population is on the rise. It has been increasing by 2 percent yearly from 1999 to 2006, and a continual increment is predicted [1]. The incidence of hip fractures worldwide is estimated to be 2.6 million in 2025 and 4.5 million by 2050 [2]. It is important to note that the population is ageing. On initial presentation, a significant proportion of patients with hip fractures have other associated medical co-morbidities. Surgical intervention is the mainstay treatment for most patients. Comprehensive care is provided by multidisciplinary team approach including the medical team to optimise the patient medically prior to surgery, as required, to improve patients' outcomes. Delay to surgery has been associated with increased morbidity and mortality in hip fracture patients.

In our department, all hip fracture patients with newly diagnosed cardiac murmur on auscultation on admission had a pre-operative echocardiogram based on NCEPOD [3] report 2001. It recommended that 'whenever possible the anaesthetist of a patient with aortic stenosis should obtain a preoperative echocardiogram of the aortic valve'. Moreover, the NCEPOD also recommended invasive monitoring and ICU/HDU, and excellent postoperative pain control for patients with aortic stenosis. The aim of our audit was to assess the impact of obtaining a pre-operative echocardiogram on the management of hip fracture patients in our unit.

## Materials and methods

We undertook a retrospective audit of hip fracture patients admitted to our district general hospital between June 08 and June 09. There were 349 (N) hip fracture patients admitted during that period. We obtained the details of all echocardiograms performed by the cardiology department for our hip fracture patients. There were 29 patients (echo group), who had an echocardiogram as part of their pre-operative work-up. A computer generated randomised sample of 40 patients was generated from the remaining 320 patients, 'non-echo' group. Demographic and clinical data was obtained from medical records and the Hospital Information Support System (HISS). We looked at delay to surgery, length of stay and mortality rates between the 'echo' and 'non echo' groups. The groups were compared using Student's t test. Approval was obtained locally from the Sunderland Royal Hospital clinical governance department for this project.

## Results

**Figure 1:**
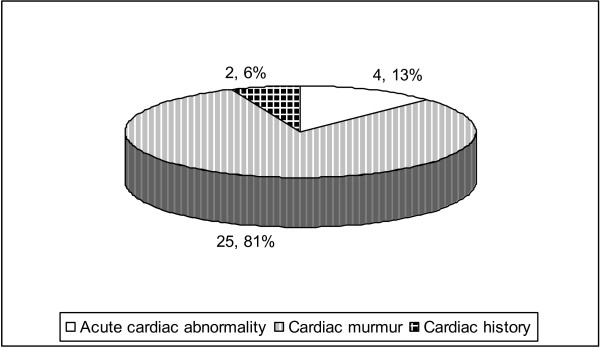
**Pie chart shows the indication for requests of echocardiogram**.

The 'echo' and 'non echo' groups were age matched (Table 1). The gender distribution was as follows: 4 males, 25 females in the 'echo' group compared to 9 males and 31 females in the 'non echo' group (Figure 1).

**Table 1:** Demographic details of patients

Patient demographics	Echo Group	Non echo Group
Number of patients	29	40
Mean age +/- SD	85.2+/- 7.7	85.0 +/- 6.6
Gender (Male/female)	4 Males	9 Males
	25 Females	31 Females

The indication for requesting a pre-operative echocardiogram was an acute cardiac abnormality in 4 cases. 25 patients had echocardiogram for no new cardiac problem (indication being cardiac murmur in 23 patients and extensive cardiac history in 2 cases). All 23 patients had newly diagnosed cardiac murmurs, and did not have an echocardiogram prior to this episode of hospital admission. The 2 patients with extensive cardiac history had previously had an echocardiogram about 2 and 3 years respectively prior to sustaining the hip fracture. The pre-operative echocardiogram in one patient showed no significant changes compared to the echocardiogram previously done. The other patient with extensive cardiac history had significant changes in the echocardiogram; this patient had medical input from the cardiologist and was referred to a specialist unit electively for a specialist opinion regarding heart valve replacement.

**Figure 2:**
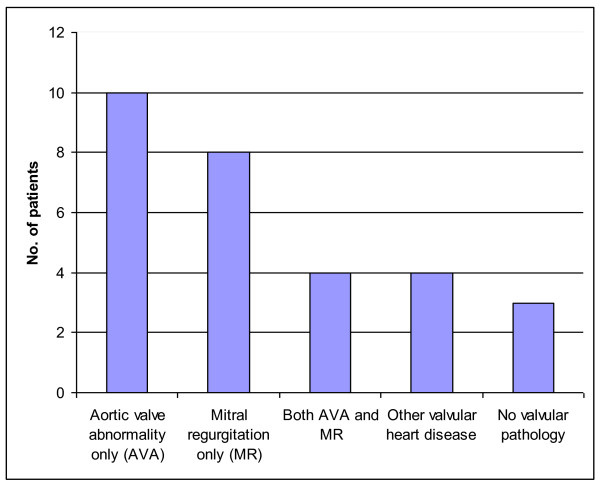
**The bar-chart shows the results of echocardiography**.

14 patients were found to have an aortic valve abnormality, out of which there were 1 case of mild aortic stenosis, 2 cases of severe aortic stenosis and 1 case of critical aortic stenosis. Aortic sclerosis and aortic regurgitation were the other aortic valve abnormality found. 10% of the patients who underwent echocardiography had no valvular pathology (Figure 2).

Cardiology opinion was sought in 5 cases. No patient required any cardiac intervention pre-operatively.

**Figure 3:**
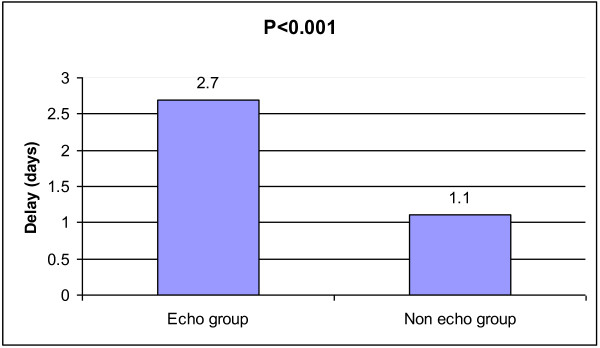
**Bar chart illustrates significant delay to surgery between the 2 groups**.

The pre-operative echocardiogram was helpful to the anaesthetic management of the patients. It aided the anaesthetist in administering a safe anaesthesia to the patients in our unit. 13 patients had surgery under general anaesthesia, out of which 8 patients had an aortic valve abnormality only, 4 patients had both an aortic valve abnormality and mitral regurgitation, and 1 patient had severe mitral regurgitation. 14 patients had spinal anaesthesia. 1 patient had peripheral nerve blocks and sedation. All the patients underwent surgery uneventfully (Figure 3).

Patients having pre-operative echo had significant delay to surgery (mean 2.7 days, range 0-6 days) compared to 'non-echo' group (mean 1.1 days, range 0-3 days), (p < 0.001).

There was no significant difference in length of stay between the echo group (mean 16.7 days) compared to 15.4 days in the 'non echo' group (p = 0.14). We found no significant difference in mortality at 30 days between the 2 groups (p = 0.41). There were 3 cases of deaths in the 'echo' group at 30 days. One patient with severe aortic stenosis was very high risk for anaesthesia. The anaesthetist and surgeon discussed with the patient and family about the benefits and risks of surgery, and the patient chose not to undergo surgery. The patient died at 7 days due to cardiac cause. There was a case of Clostridium related death post-operatively in a patient with no valvular pathology. Another patient with aortic regurgitation died at 14 days due to non ST elevation myocardial infarction. There were 2 cases of death due to pneumonia in the 'non echo' group.

## Discussion

Aortic stenosis is the most common form of acquired valvular heart disease in developed countries; it is estimated to occur in 2-4% of the population aged over 65 years old [4]. It is not uncommon to have a hip fracture patient with a cardiac murmur, or even aortic stenosis. It appears that the combination may be associated with a higher morbidity and mortality rate.

Pre-operative cardiac testing has its place in the elective setting. In the emergent situation, the clinician needs to evaluate the risk incurred by waiting for the cardiac testing when compared to the risks associated with the delay to surgery. In a recent national survey of anaesthetists on the perioperative management of hip fracture patients with a previously undiagnosed heart murmur, the responses were mixed. Most anaesthetists would ask for a pre-operative echocardiogram in the presence of suspicious signs or symptoms, whereas 19.8% would be prompted to use invasive monitoring without an echocardiogram [5].

According to Parker et al, regional and general anaesthesia produce comparable results for hip surgery outcome [6]. Pellikka et al [7] reported that surgery may not pose any additional risks for patients with aortic stenosis. There was no report of statistically significant difference in anaesthetic management of hip fracture patients with different severity of aortic stenosis compared to patients without aortic stenosis by Adunsky et al [8]. McBrien et al [9] reported a trend towards general anaesthesia versus spinal anaesthesia in hip fracture patients with varying severity of aortic stenosis; invasive monitoring was also used in some patients. Whilst the pre-operative echocardiogram did not alter the orthopaedic management of the patients, apart from one patient who declined surgery; it appeared helpful in the anaesthetic management. In our patients with aortic stenosis, 1 patient with severe aortic stenosis underwent surgery with peripheral nerve blocks plus sedation, 1 patient with critical aortic stenosis had general anaesthesia, 1 patient with mild aortic stenosis had spinal anaesthesia, and 1 patient with severe aortic stenosis refused surgery. Invasive monitoring was used in none of the patients.

10% of patients in the 'echo' group had no valvular heart disease. Interestingly, a recent study showed that a cardiac murmur suggestive of aortic stenosis, diagnosed on admission in 908 hip fracture patients was confirmed by echocardiography in only 30% of cases [9]. Abnormal auscultatory findings can lead to unnecessary referral for echocardiogram.

There is controversy regarding the acceptable delay for surgery in hip fracture patients. A recently published guideline advocated timely and co-ordinated multi-disciplinary care and operative intervention at 36 hours for improved outcomes in hip fracture patients [10]. Early surgery is associated with less pain, improved functional outcome, shorter length of stay in hospital and post-operative complications such as: deep venous thrombosis, pulmonary embolism and pneumonia [11-13].

However, optimisation of hip fracture patients with active medical co-morbidities is also important [14,15]. A systematic review by Shiga et al [16] reported that hip fracture surgery delay beyond 48 hours increased the odds of 30-day mortality by 41% and 1 year mortality by 32%. They commented that due to methologic limitations, definitive conclusions could not be drawn. Another study reported that there was no association between delay in hip fracture surgery and mortality after adjustment for medical co-morbidities [17]. There was no significant difference in the length of stay of the hip fracture patients in the 'echo' compared to the 'non echo' group. We found no significant differences in mortality rates at 30 days in the 'echo' compared to the 'non echo' group.

## Conclusion

The exact answer to timing of hip fracture surgery is uncertain. Careful patient selection for pre-operative echocardiography is important to avoid unnecessary delay to surgery. Based on multidisciplinary care, a selected group of hip fracture patients with cardiac murmur will have an echocardiogram pre-operatively, local guidelines are underway. The clinical audit was a useful tool for highlighting the need for resource allocation to accommodate the demand for pre-operative echocardiogram in hip fracture patients. We have developed departmental guidelines for expediting echocardiogram requests in hip fracture patients with cardiac murmur. A liaison has been established with our cardiology department for targeted echocardiogram in these patients. Further study is required to determine the cost-effectiveness and benefits of such approach.

## Competing interests

The authors declare that they have no competing interests.

## Authors' contributions

PJ was the chief investigator, developed design and methods, collected the data and performed data analysis, drafted the manuscript and is responsible for the final approval of the manuscript. RK and SJ contributed to the methodology and discussion. IT identified the topic as a subject of current interest. PD contributed to the discussion. All authors have read and approved the final manuscript.
